# Preliminary report on the landslide early warning on August 20, 2021, in Nangqian County, Qinghai Province, China

**DOI:** 10.1038/s41598-022-13353-4

**Published:** 2022-06-13

**Authors:** Xiangpeng Wang, Kunpeng Wang, Fanqiang Lin, Kai Guo

**Affiliations:** 1 Chengdu University of Technology, Chengdu, 610059 Sichuan China; 2 Chengdu University of Technology Boda Engineering Co., Ltd, Chengdu, 610059 Sichuan China

**Keywords:** Limnology, Natural hazards

## Abstract

Based on an analysis of the formation mechanism, stability state and development trend, this paper focuses on monitoring key indicators, such as surface deformation and rainfall, to realize the early warning of landslides and provide some guidance for landslide disaster prevention and avoidance in the alpine mountainous area of Nangqian, Qinghai Province. The landslide of the Baima Elin Monastery has the topographic and geomorphological conditions of landslide formation and has the basic characteristics indicating that it can be influenced by rainfall and climate, so a landslide warning should be comprehensive. At the same time, the landslide is currently in an unstable state, and the seasonal freeze–thaw cycle will cause it to become more unstable. Based on macroscopic signs, it is predicted that the deformation of the Baima Elin Monastery landslide will continue to intensify without abatement, the stability will be further reduced after winter, developing in an unfavorable direction, and engineering measures should be taken as soon as possible to manage the landslide.

## Introduction

**Figure 1:**
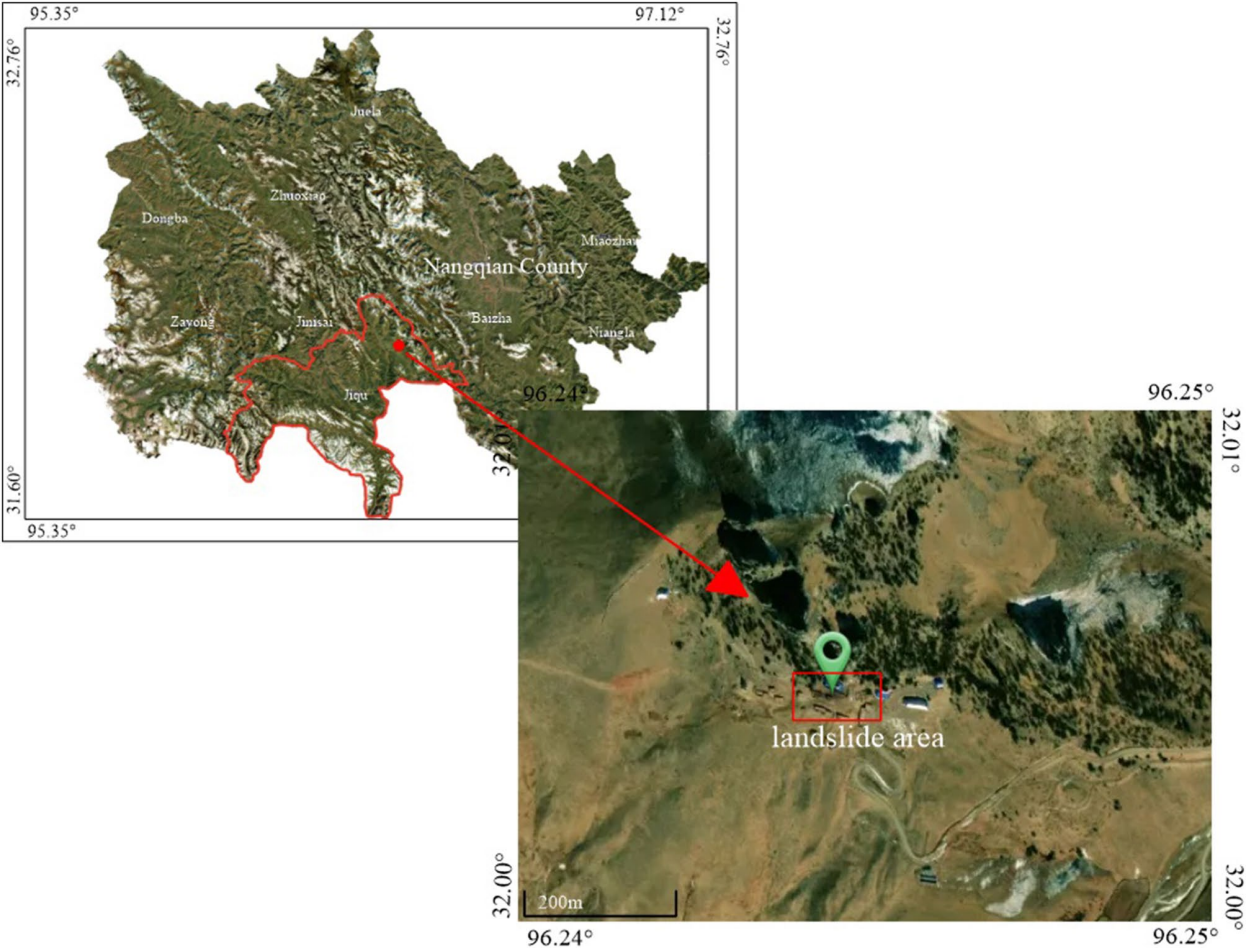
Aerial satellite image of the landslide (download using BIGEMAP GIS).

Nangqian is located on the southeastern Tibetan Plateau and stretches over a vast area from the southern Kunlun Mountains to the northern Tanggula Mountains. The county is the southern gate of Qinghai Province and is also a major pastoral region. Within the region, the terrain has high altitudes, deep gaping valleys and continuous tall mountains. Since August 10th, 2021, an increase in rainfall has been witnessed in the Baima Elin Monastery area of Jiqu Township, Nangqian County, Yushu, Qinghai Province. The geological location is 96°14′51.00″ longitude and 32°00′42.12″ latitude, as shown in Fig. 1. On August 19th, the rainfall per hour reached 8 mm and totaled 100 mm for the day. In addition, a geological disaster early warning was given at 4:00 AM on August 20th.

**Figure 2:**
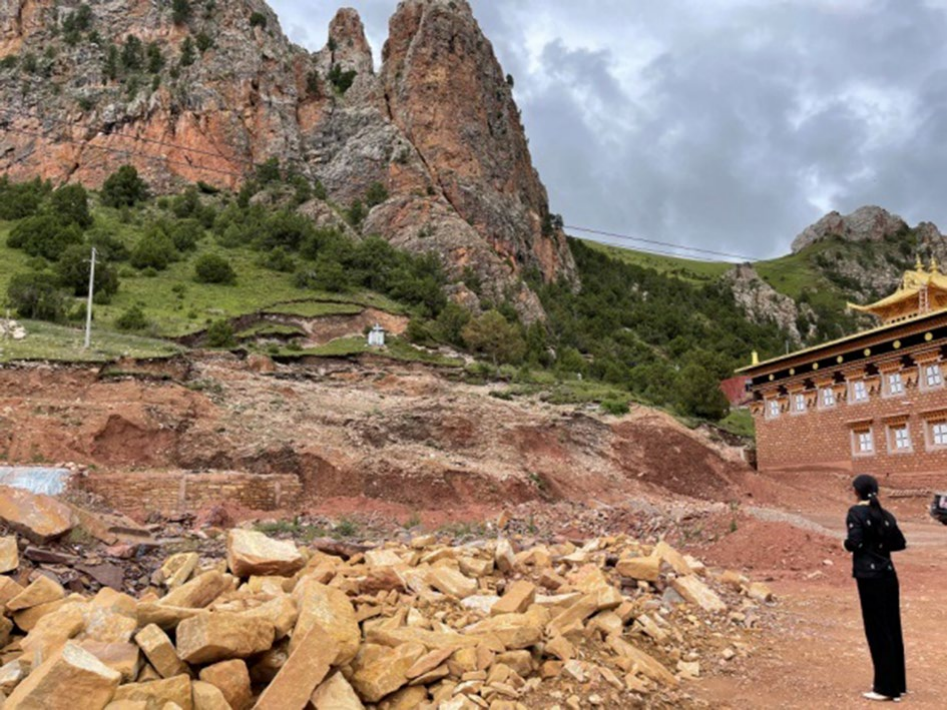
Panorama of the landslide.

The landslide reached 25 m^3^, causing different scales of damage to the monk's retreat and the wall of the great hall, but the landslide did not cause direct economic losses and casualties. The panorama of the landslide is shown in Fig. 2. After the landslide, at 11:00 AM on August 20, 2021, technicians of the Qinghai Coal Geo-engineering Co. LTD visited the site and investigated the landslide. The landslide is approximately 35 m long, 85 m wide, 4 m thick, 1.2 × 10^4^ m^3^ in scale and 5.2 × 10^4^ m^3^ in volume. At present, the landslide's extensional crack along its back edge tends to widen and deepen. Heavy rainfall and earthquakes may cause local landslides or collapse disasters, which pose a threat to the monastery.

In contrast to the previous red bed soft rock landslide and collapsible loess landslide, this landslide is in a high-altitude area. Its geomorphic and geological conditions are affected by seasonal freeze–thaw cycles and rainfall, which have typical representative and regional characteristics. The Baimaolinsi landslide is directly affected by continuous rainfall. Through the successful early warning of landslides and by comparing other similar landslides in the study area, this paper summarizes the quantitative relationship between single-day rainfall, the relationship between continuous rainfall accumulation and location, and the occurrence of landslides, thereby providing a reference case for landslide early warnings on the Qinghai-Tibetan Plateau.

## Geological and morphological setting

**Figure 3:**
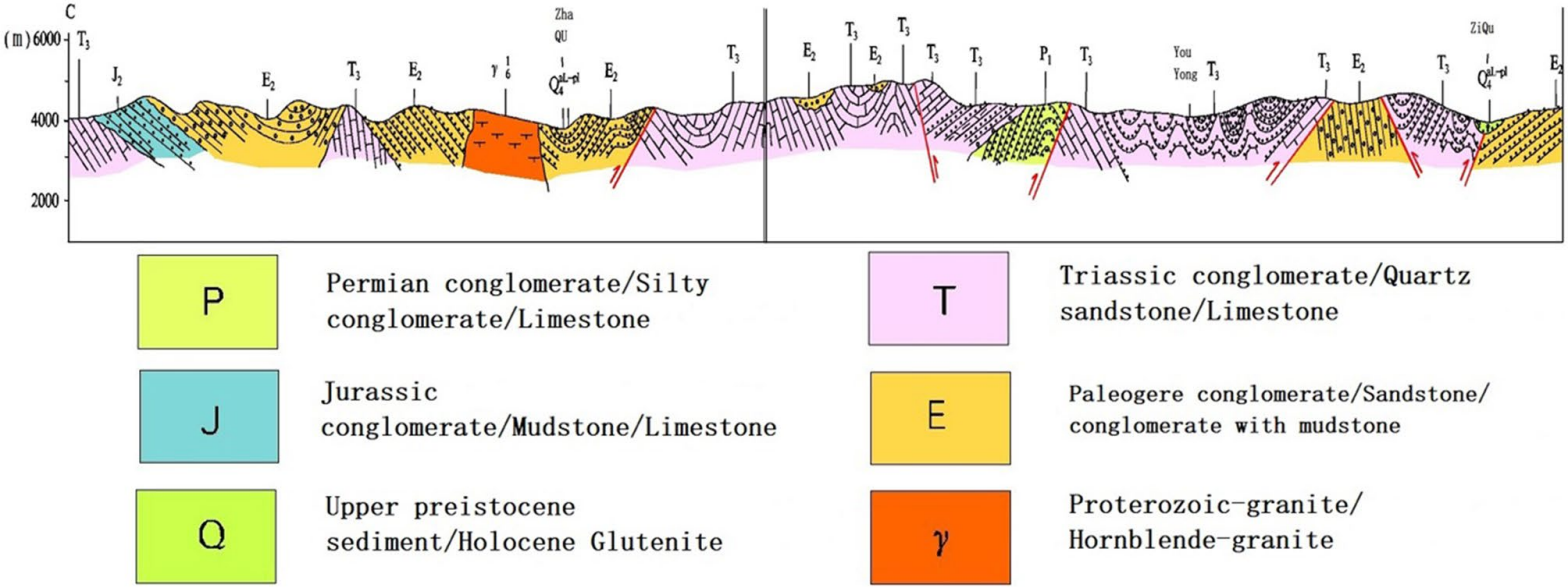
Geological profile (this figure is taken from 7 references: <China Geological Survey, Ministry of land and resources. Detailed investigation of geological hazards in Qinghai>).

The landslide is located on the northern slope of Shanrong village, Jiqu Township, Nangqian County. It is on the front edge of a low mountain in an erosional structure, and it generally slopes 25°–35° and has a relative height difference of 90 m. The original slope in the survey area is Neogene mudstone, which has a formation mechanism. The rock quality is very soft and weakly expanding; the rock is easy to soften and disintegrates when in contact with water, and it has poor weather resistance^1–3^. The surface of the exposed formation in the landslide area is Quaternary broken gravel^4,5^. The lithology is yellow–brown conglomerate, gray–purple conglomerate, medium- to fine-grained quartz sandstone, quartz siltstone, purple–red mudstone, and gray–white gypsum; the stratigraphic section is shown in Fig. 3^6^. The back edge of the landslide is located on the south side of the steep sinking bump under the landslide, which is a potential landslip area, and this area has elevations of 3658 to 3670 m. The slope is rather complete, no obvious signs of deformation are present, and the average slope is 22 degrees. The top of the slope is covered by gravels, the gravels are angular, and the particle sizes range from 0.1 to 0.5 m. From the top of the slope to the steep part, vegetation coverage gradually increases, and the coverage ranges from 15 to 55%.

The survey information indicates that mixed-structure powdered clay is distributed on the landslide slope in the survey area, the thickness of the layer is uneven, the structure is messy and includes gravel and mud rock blocks, and the mechanics are poor^7^. Miscellaneous fill is distributed mainly in the exploration area. The artificially excavated urban underground drainage system and leveling site is located at the foot of the landslide slope, and the impurities include concrete and bricks. The soil is slightly wet and has a loose structure and poor mechanics.

## Characteristics of the landslide

**Figure 4:**
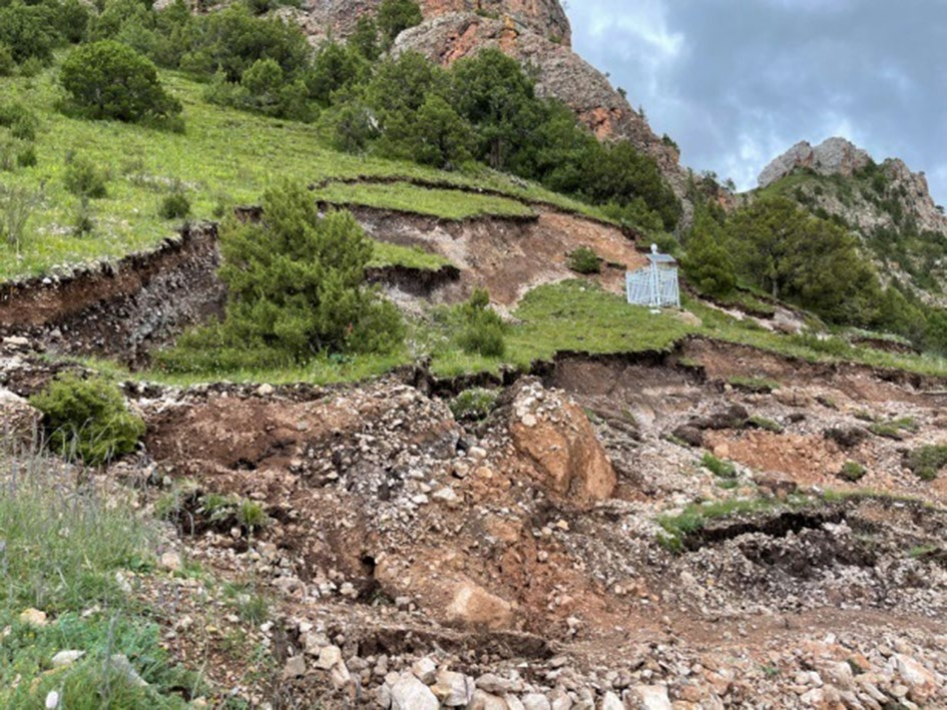
Tensile cracks in the trailing edge (2015.07).

On-site investigations reveal that the landslide is a small moving earthen landslide with a trailing edge elevation of 3704 m, leading edge elevation of 3683 m, landslide height of 21 m, slope of 45 degrees, and landslide sliding direction of 155 degrees. The landslide is in the stage of peristaltic deformation, and field inquiries and investigations reveal that the landslide's expansion cracks formed in two stages, which were directly related to rainfall. The first stage was in mid-July 2015; on mainly the north side of the landslide's back edge, two tensile cracks formed, which were approximately 3–5 m and had crack widths of 0.2–0.3 m but less than 0.3 m and a maximum observed depth of 0.5 m. It was a semicircular distribution, as shown in Fig. 4.

**Figure 5:**
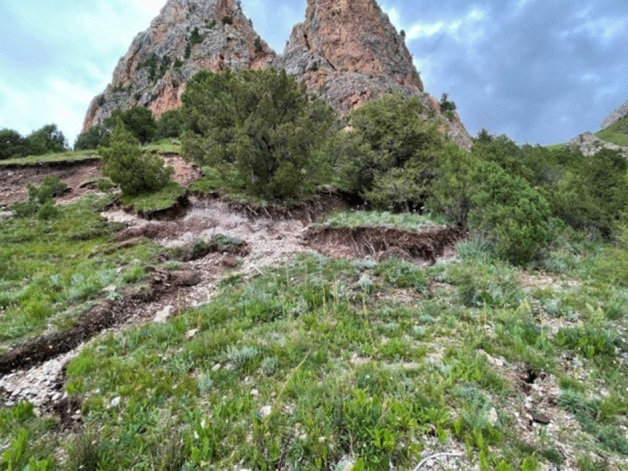
Landslide tensile cracks (2021.08).

The second landslide occurred at 4:00 AM, August 20th, 2021. Ten tensile cracks formed on the upper slope; their lengths ranged from 2 to 8 m, widths ranged from 0.15 to 0.5 m, total sinking depths ranged from 1 to 3 m, and visible depths ranged from 0.5 to 1.5 m. The cracks were in an east–west orientation, as shown in Fig. 5.

## Process monitoring

**Figure 6:**
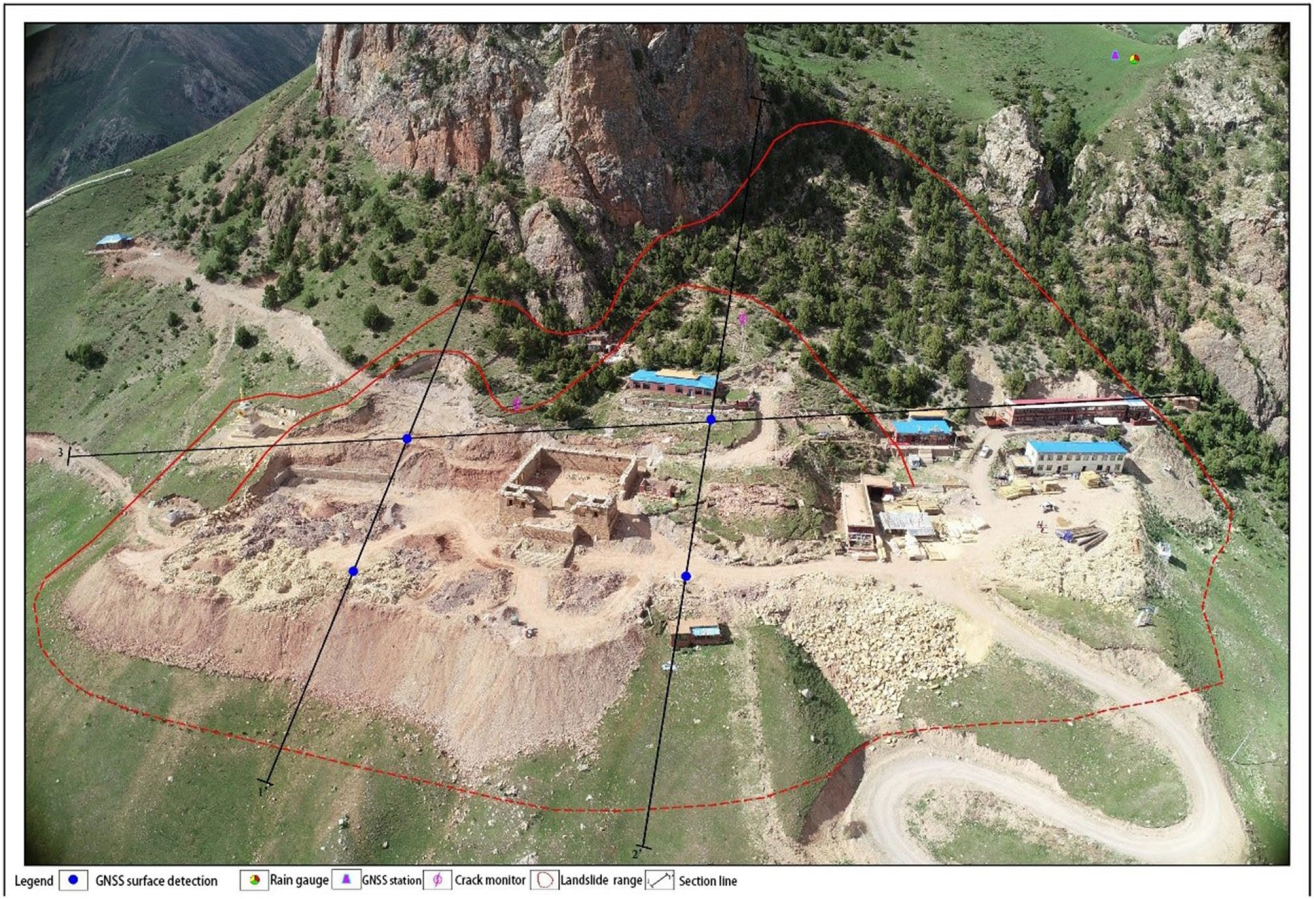
Landslip monitoring setup.

**Figure 7:**
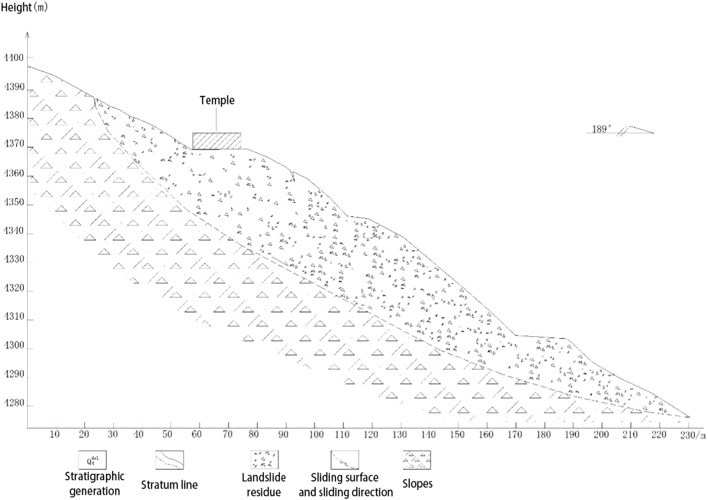
Geological disaster profile 2–2.

For the prevention of landslide geological disasters, the local government jointly undertook prevention research with the State Key Laboratory of Geohazard Prevention and Geoenvironment Protection (Chengdu University of Technology) and Qinghai China Coal Geological Engineering Co., Ltd. in January 2021. The disaster location, four earth movement monitors, one GNSS base, two fissure meters, and one rain gauge were established, according to Fig. 6, and the geological disaster 2–2 survey line profile is shown in Fig. 7.

The surface displacement meter/GNSS is a space-based radio navigation and positioning system that provides all-weather three-dimensional coordinates, speed and time information at any location on the earth's surface or near-earth space by using the pseudo-range, ephemeris, satellite launch time and user position clock difference of a group of satellites. The parameters are as follows: relative positioning accuracy in the horizontal direction: ± (5 mm + 1 ppm); RMS in the vertical direction: ± (10 mm + 1 ppm); RMS sampling interval: 1 h; crack meter measuring range: 0–50/100//200/500 cm; measurement accuracy: ± 0.1% F·S; and sampling interval: 1 h.

The tipping bucket rain gauge measures the natural rainfall and converts the rainfall into a digital information output in the form of a changing value to meet the needs of information transmission, processing, recording and display. The 0.2 mm tipping bucket rain gauge adopted this time uses 2/3/4G transmission. The parameters are as follows: measurement range of the system: 0–8 mm/min; resolution: 0.2 mm; and sampling interval: 1 h.

## Development and forecast

**Figure 8:**
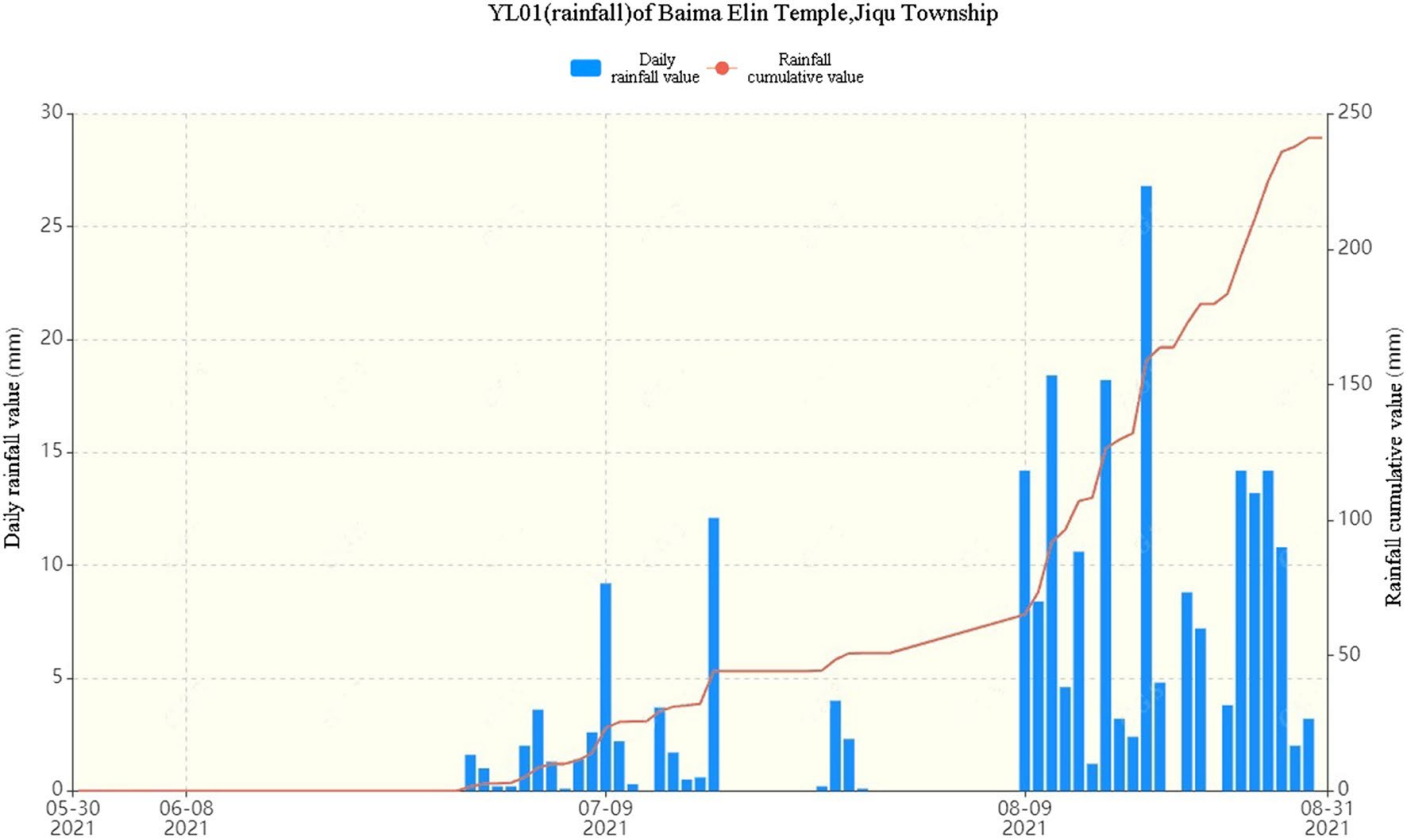
Rainfall on a single day and accumulated rainfall from May 31st to August 31st, 2021.

**Figure 9:**
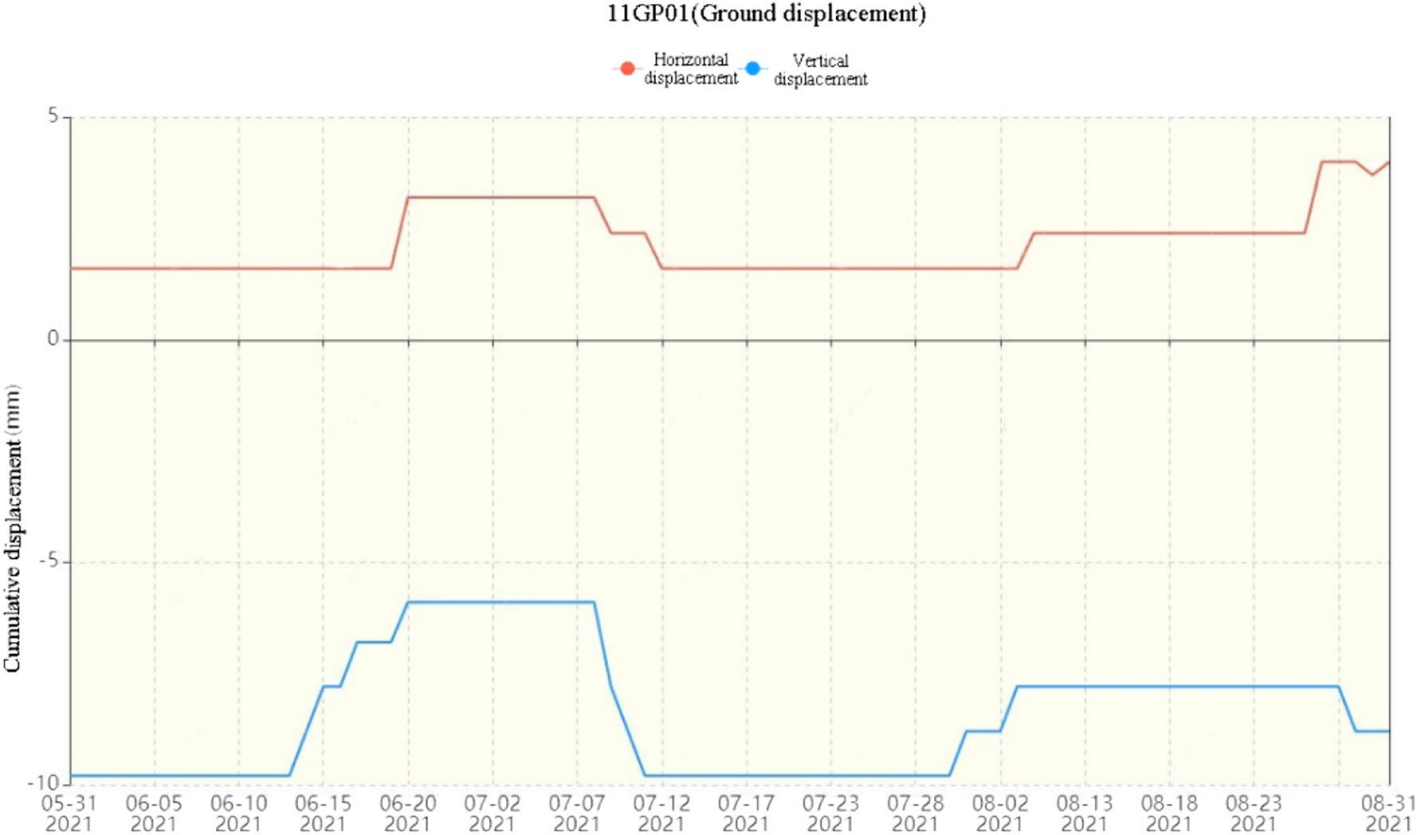
Accumulated horizontal and vertical movement from May 31st to August 31st, 2021.

The rain gauge data of the Baima Elin Monastery of Jiqu Township show that starting on August 10th, the rain gauge data increased, and the rainfall reached 8 mm per hour on August 19th, with an accumulated rainfall of approximately 100 mm (the rainfall capacity is shown in Fig. 8). Continuous heavy rainfall caused rain to soak into the loose layer and existing cracks, thereby increasing the weight of the slope and lowering the shear strength, which is the direct cause of landslides^4,5^. At 4:00 AM, August 20th, in the Baima Elin Monastery, Jiqu Township, Nangqian County, Yushu, Qinghai Province, the 02GP01 surface displacement meter triggered a single parameter warning, the 11GP01 surface displacement meter triggered an orange alert (surface movement data are shown in Fig. 9), and the landslide observation system triggered an orange alert; an orange alert means that the slope is in the stage of accelerated deformation, and the probability of a landslide within a few days is very high.

A comparison of Figs. 8 and 9 shows that when continuous rainfall occurs, the horizontal displacement and vertical displacement of the crack increase, and the displacement of the crack changes stably within a certain period of time. The stable growth of the single-day displacement recorded by the displacement meter before the landslide occurred lasted approximately 10 days, the stable value of the vertical displacement was approximately 2.5 mm, and the stable value of the horizontal displacement was approximately 7.6 mm. The length of the stabilization period with increasing displacement is basically proportional to the time required for the rainwater to completely soak the rock mass until the rock mass becomes saturated with water. According to the statistics of multiple landslide points in the study area, landslides of different sizes occur when the daily rainfall reaches more than 22 mm and the cumulative continuous rainfall reaches more than 112 mm.

## Conclusion

The landslip at the Baima Elin Monastery in Jiqu Township was mainly caused by continuous heavy rainfall and favorable landforms, strata, lithologies and regional tectonic conditions. Rainfall is the main factor that induces geological disasters. The infiltration of rainwater reduces the shear strength of the soil mass, increases the self-weight of the rock and soil masses, increases the sliding force, and reduces the stability coefficient of the slope, resulting in slope sliding. The successful early warning effectively ensured the safety of temple monks and reduced economic losses. Before the construction of the project, the regional geological disasters should also be evaluated, and the areas that are prone to geological disasters should be avoided as much as possible to prevent accidents and reduce the loss of capital investment. At the same time, the government should strengthen the existing potentially dangerous slopes to prevent the recurrence of disasters and effectively prevent geological disasters.
